# Genome resources and whole genome resequencing of *Phytophthora rubi* isolates from red raspberry

**DOI:** 10.3389/fpls.2023.1161864

**Published:** 2023-06-29

**Authors:** Sanjib Sapkota, Rishi R. Burlakoti, Mark Lubberts, Kurt Lamour

**Affiliations:** ^1^ Agassiz Research and Development Centre, Agriculture and Agri-Food Canada, Agassiz, BC, Canada; ^2^ Summerland Research and Development Centre, Agriculture and Agri-Food Canada, Summerland, BC, Canada; ^3^ Department of Entomology and Plant Pathology, University of Tennessee, Knoxville, TN, United States

**Keywords:** clonal, genome, genetic diversity, *Phytophthora rubi*, raspberry, heterozygosity

## Abstract

*Phytophthora rubi* is a primary causal agent of Phytophthora root rot and wilting of raspberry (*Rubus idaeus* L.) worldwide. The disease is a major concern for raspberry growers in Canada and USA. To date, no information is available on genomic diversity of *P. rubi* population from raspberry in Canada. Using a PCR-free library prep with dual-indexing for an Illumina HiSEQX running a 2x150 bp configuration, we generated whole genome sequence data of *P. rubi* isolates (*n* = 25) recovered during 2018 to 2020 from nine fields, four locations and four cultivars of raspberry growing areas of British Columbia, Canada. The assembled genome of 24 isolates of *P. rubi* averaged 8,541 scaffolds, 309× coverage, and 65,960,000 bp. We exploited single nucleotide polymorphisms (SNPs) obtained from whole genome sequence data to analyze the genome structure and genetic diversity of the *P*. *rubi* isolates. Low heterozygosity among the 72% of pathogen isolates and standardized index of association revealed that those isolates were clonal. Principal component analysis, discriminant analysis of principal component, and phylogenetic tree revealed that *P. rubi* isolates clustered with the raspberry specific cultivars. This study provides novel resources and insight into genome structure, genetic diversity, and reproductive biology of *P rubi* isolated from red raspberry. The availability of the *P. rubi* genomes also provides valuable resources for future comparative genomic and evolutionary studies for oomycetes pathogens.

## Introduction

*Phytophthora rubi*, the primary causal agent of Phytophthora root rot and wilting (PRRW), is the most destructive pathogen of red raspberry (*Rubus idaeus* L.) in the Pacific Northwest regions of Canada (Sapkota et al., 2022a; Sapkota et al., 2022b) and the United States (Stewart et al., 2014; Weiland et al., 2018) and other raspberry producing areas worldwide (Duncan et al., 1987; Wilcox et al., 1993; Wilcox and Latorre, 2002; Jennings et al., 2003; Sapkota et al., 2022b). This pathogen poses a serious economic impacts to raspberry producers by reducing plant vigor, lifespan, and yield (Burlakoti and Dossett, 2020; Sapkota et al., 2022b). In addition with *P. rubi*, other species of *Phytophthora* such as, *P. gonapodyides*, *P. cryptogea*, *P. citricola*, and *P. megasperma* were also sporadically reported to infect raspberry (Wilcox, 1989; Wilcox and Latorre, 2002; Sapkota et al., 2022b; Burlakoti et al., 2023).

*Phytophthora rubi* is considered as a homothallic species and produces both sexual and asexual spores (Tabima et al., 2018; Abad et al., 2023). Mycelia and oospores in infected tissue and soil are major sources of inocula. The mycelia and sporangia can directly infect the basal stem and root tissues of plants. Sporangia also produces motile zoospores for infection in presence of high soil moisture. Sporangia and zoospores can travel in soil *via* water allowing *P. rubi* to migrate within and among fields. The dispersal and germination of these spores are favored by high soil moisture due to either rain or irrigation and average temperatures of 15 to 20°C (Graham et al., 2021). The pathogen spores colonizes root tissues, interferes water and nutrient movement in vascular system and develop dark-reddish brown lesions on roots and crown. As the disease progresses, foliage symptoms, such as necrosis, scorching, and chlorosis of leaves, and wilting of branches appears (Sapkota et al., 2022b).

In the past decades, genomes of multiple species of *Phytophthora*, such as *P. infestans* (Haas et al., 2009), *P. sojae*, *P. ramorum* (Tyler et al., 2006), *P. capsici* (Lamour et al., 2012), and *P. fragariae* (Gao et al., 2015) were sequenced. Recently, a draft genome of one isolate of *P. rubi* of raspberry was reported from the western USA (Tabima et al., 2017). However, whole genome sequencing resources of isolates of *P. rubi* from Canada are not available. The genetic structure of *P. rubi* isolated from raspberry has been analyzed in western USA using amplified fragment length polymorphism, and genotyping by sequencing (Stewart et al., 2014; Tabima et al., 2018). Both studies showed a low genetic diversity and low differentiation among *P. rubi* population. In addition, single nucleotide polymorphisms (SNPs) obtained from high throughput sequencing have recently been utilized to infer population structure, and its reproductive strategies in many species of *Phytophthora* such as *P. capsici* (Castro-Rocha et al., 2016; Siegenthaler et al., 2022), *P. pluvialis* (Brar et al., 2018), and *P. lateralis* (Quinn et al., 2013). High throughput sequencing is progressively becoming less expensive and data provide useful tools to explore genetic diversity and potentially aid in identifying the areas of diversity driving host varietal preference.

Although *P. rubi* has been reported as a major causal agent of PRRW in raspberry growing areas in Canada (Sapkota et al., 2022a; Sapkota et al., 2022b), no information is available on genetic structure and diversity of *P. rubi* population. Therefore, the objectives of this study were to: i) generate genomic resources of *P. rubi* using whole genome sequencing, and ii) study the genome structure and genetic diversity of *P. rubi* isolates from raspberry.

## Materials and methods

### *Phytophthora rubi* isolates

Isolates in this study were selected from the large culture collection from our previous study on raspberry root rot and wilting complex of raspberry on BC (Sapkota et al., 2022a). These isolates (*n* = 25) were collected over multiple years (2018 to 2020) from nine fields, and four cultivars of raspberry (**Table 1**). The detail of sampling, isolation and identification of the pathogen was described in (Sapkota et al., 2022a). In brief, pathogen isolates were obtained from infected root and cane tissues of raspberry using CMA-PARPH (corn meal agar medium amended with pimaricin, ampicillin, rifampicin, pentachloronitrobenzene and hymexazol). The hyphal tips of each isolate grown on CMA-PARPH were transferred to 20% clarified V8PAR for further morphological characterization. Colony and sporangia morphology was used for morphological characterization. For the molecular identification, isolates were grown on V8PAR media with cellophane on the surface for 10 to 13 days. Mycelia were then collected, freeze-dried, and ground using a magnetic MixerMill bead beating device (Qiagen). High quality genomic DNA was extracted using the MagMAX kit (ThermoFisher Scientific Inc.) following the manufacturer’s instructions. Pathogens were identified using multiplex targeted-sequencing with degenerate primers of three nuclear genes: heat shock protein90, elongation factor 1 alpha and beta tubulin as described in (Sapkota et al., 2022a).

**Table 1:** Summary of comparative assembly statistics and gene predictions for genome resequencing of 24 isolates of *Phytophthora rubi*.

Isolate	Cultivar	Year	Field	Location	Total size (bp)	Mean coverage	Number of scaffolds	Scaffold N50 (bp)	Total predicted genes	Total predicted proteins	Repeat content (%)
BC83	‘Rudi’	2019	F6	Abbotsford	64,110,000	394.03	9,231	13,640	16,561	16,719	16.26
RB2	‘Rudi’	2020	F6	Abbotsford	66,670,000	356.04	7,768	15,388	17,831	18,005	19.19
RB3	‘Rudi’	2020	F6	Abbotsford	65,870,000	306.85	7,757	15,231	16,856	17,022	19.50
RB4	‘Rudi’	2020	F6	Abbotsford	65,850,000	355.31	7,700	15,256	16,889	17,067	19.08
RB42	‘Rudi’	2020	F19	Abbotsford	71,160,000	541.13	7,389	17,132	17,412	17,553	22.40
RB5	‘Rudi’	2020	F6	Abbotsford	66,370,000	295.91	8,242	14,436	17,347	17,514	19.12
RB6	‘Rudi’	2020	F6	Abbotsford	66,220,000	361.18	7,994	14,939	17,714	17,892	18.48
RB7	‘Rudi’	2020	F6	Abbotsford	66,630,000	354.17	8,035	14,708	17,019	17,175	20.01
RB8	‘Rudi’	2020	F6	Abbotsford	66,530,000	315.31	8,182	14,411	17,192	17,347	19.80
BC82	‘Chemainus’	2019	F23	Chilliwack	67,890,000	373.70	9,959	11,698	18,956	19,141	19.20
RB14	‘Chemainus’	2020	F9	Abbotsford	67,230,000	122.66	9,199	12,315	17,930	18,114	18.95
RB16	‘Chemainus’	2020	F9	Abbotsford	70,950,000	389.95	7,359	16,970	18,335	18,494	22.44
RB17	‘Chemainus’	2020	F9	Abbotsford	59,450,000	52.04	11,143	8,822	17,734	17,955	12.41
RB18	‘Chemainus’	2020	F9	Abbotsford	64,420,000	175.65	7,850	16,369	17,176	17,358	18.19
RB19	‘Chemainus’	2020	F9	Abbotsford	64,500,000	169.81	7,826	16,047	16,821	17,013	17.65
RB20	‘Chemainus’	2020	F9	Abbotsford	64,220,000	165.69	7,782	16,240	17,861	18,040	17.64
RB27	‘Chemainus’	2020	F17	Abbotsford	70,860,000	664.45	7,203	17,699	18,376	18,575	22.48
RB58	‘Chemainus’	2020	F23	Chilliwack	62,580,000	187.37	9,079	13,444	16,792	16,987	16.39
RB70	‘Chemainus’	2020	F25	Agassiz	63,910,000	146.58	8,306	14,937	16,695	16,890	17.00
RB83	‘Chemainus’	2020	F28	Abbotsford	64,090,000	137.49	8,235	15,349	16,739	16,925	17.78
BC3	‘Meeker’	2018	F7	Abbotsford	62,060,000	389.57	10,532	10,921	19,442	19,601	14.24
BC6	‘Meeker’	2018	F7	Abbotsford	66,710,000	397.37	9,848	11,783	18,549	18,730	18.30
RB13	‘Meeker’	2020	F7	Abbotsford	71,060,000	394.25	7,340	17,189	17,450	17,608	21.66
BC68	‘Cascade Delight’	2019	F22	Delta	63,700,000	389.86	11,070	11,351	17,675	17,868	15.09

### Library preparation and genome sequencing

Genomic DNA of the pathogen was extracted as described in the previous section. Approximately, one µg of high quality genomic DNA was sheared to a size in between 250 and 500 bp with a Covaris M220 focused ultrasonicator (Covaris, Inc., Woburn, MA, U.S.A.). After shearing, fragmented DNA was ligated with dual-indices using a KAPA Hyperprep PCR-free library kit (Roche) for Illumina according to the manufacturer’s instructions. Libraries were loaded and sequenced on an Illumina HiSEQX next-generation sequencing device running a 2 x 150 bp paired-end configuration at Admera Health LLC (Plainfield, NJ) according to the manufacturer’s directions. Raw paired-end sequence data for each sample are publicly available for download in NCBI BioProject accession number PRJNA856328.

### Alignment and variant calling

Raw sequence data was quality checked using FastQC (https://www.bioinformatics.babraham.ac.uk/projects/fastqc/). Paired-end reads were trimmed for the presence of adapters and low-quality sequences with BBDuk (https://jgi.doe.gov/data-and-tools/software-tools/bbtools/). To identify SNPs, paired-end reads were randomly subsampled to 50 million read pairs with reformat.sh (https://jgi.doe.gov/data-and-tools/software-tools/bbtools/) and then aligned to *P. rubi* reference genome (Tabima et al., 2017) using BWA-mem2 (Vasimuddin et al., 2019). SAMtools software package was used to sort, validate, and filter aligned reads (Li et al., 2009). SNPs or variants were called using Freebayes (Garrison and Marth, 2012), then filtered using VCFtools (Danecek et al., 2011) with minimum mapping quality score of 20, minimum depth of 10, and maximum depth of 300.

### Genome assembly

For whole genome assembly and annotation, a workflow was developed in the Nextflow workflow management system (Di Tommaso et al., 2017). Briefly, raw reads were quality and adapter trimmed by BBduk, then mapped to masked human and plant genomes using BBMap to remove host and contamination DNA, followed by normalization and error correction with BBNorm and Tadpole (https://jgi.doe.gov/data-and-tools/software-tools/bbtools/). The processed reads were assembled using assemblers abyss-pe v2.3.5 (Jackman et al., 2017) and Spades v3.15 (Prjibelski et al., 2020), and the assembly with the highest N50 value was selected for annotation. Assemblies were filtered with Tiara (Karlicki et al., 2022) (minimum length 1000bp) to identify potential non-eukaryotic co-isolates, and any sequences identified as bacteria or archaea were excluded from annotation. Filtered assemblies were then repeat masked with EDTA (Ou et al., 2019), and annotated with Funannotate v1.8.13 (https://github.com/nextgenusfs/funannotate), with RNAseq data SRR10207404 (Adams et al., 2020) used as transcript evidence. Gene annotation was performed using Interproscan (Jones et al., 2014) and SignalP v6 (Teufel et al., 2022), and integrated into the assembly using Funannotate. Genome completeness was evaluated using BUSCO v5.4.4 in ‘protein’ mode with the Eukaryota Odb10 gene set (Simão et al., 2015) on the predicted proteins from each genome.

### Population diversity analyses

The filtered VCF files were analyzed using the R packages vcfR (Knaus and Grünwald, 2017), poppr (Kamvar et al., 2014), data.table (Dowle et al., 2019), and adegenet (Jombart et al., 2010). To determine genetic diversity of the *P rubi* isolates, Simpson’s diversity index, λ, (Simpson, 1949), Shannon-Weiner’s genotypic diversity, H, (Shannon, 2001 and review), and Nei’s unbiased gene diversity, Hexp, (Nei, 1978) were calculated. The heterozygous variants per kb were determined to investigate the distribution of variants in pathogen population. To test the hypothesis of clonal reproduction, we estimated the standardized index of association (r̄_d_). Statistical significance of r̄_d_ test was calculated with 999 permutations of the random test. The r̄_d_ values would be expected to be zero, if isolates are mating randomly (i.e. freely recombining) (Agapow and Burt, 2001). We carried out discriminant analysis of principal components (DAPC) in the R package adegenet to show clustering among genetically related populations (Jombart et al., 2010). The advantage of the DAPC approach is that it does not make assumptions on population models or data structure. Principal component analysis (PCA) was also performed using the glpca command to display major genetic variations. A phylogenetic tree was constructed based on Bruvo’s (Bruvo et al., 2004) distance to calculate the genetic relatedness among the population of *P. rubi*. These results were plotted and visualized using the R package “ggplot2” (Wickham, 2016). We excluded one isolate (RB15) in the genome assembly since we could not identify a clear reasons for size difference of this isolates with remaining 24 isolates.

## Results

### Genome assembly

In total, whole genomes of 25 P*. rubi* isolates collected from diverse cultivars and locations were sequenced. The summary of total genome size, number of scaffolds, scaffold N50, total predicted genes, total predicted proteins, and percent repeat content of these isolates is shown in **Table 1**. The average genome size was 65,960,000 bp (range: 59,450,000 to 71,160,000 bp). The mean coverage ranged from 52 to 664×, with an average of 309×. The assembled genomes averaged 8,541 scaffolds (range: 7,203 to 11,143) with an average N50 of 14,426 (range: 8,822 to 17,668). The total number of predicted genes and protein ranged between 16,561 to 18,956 and 16,719 to 19,141 respectively. The repetitive content in *P. rubi* genome assemblies ranged from 12.41 to 22.48%. In addition, BUSCO completeness scores ranged from 92.2 to 94.2%, with an average of 93.33% (**Supplementary Table 1**).

### Population diversity analyses

**Figure 1:**
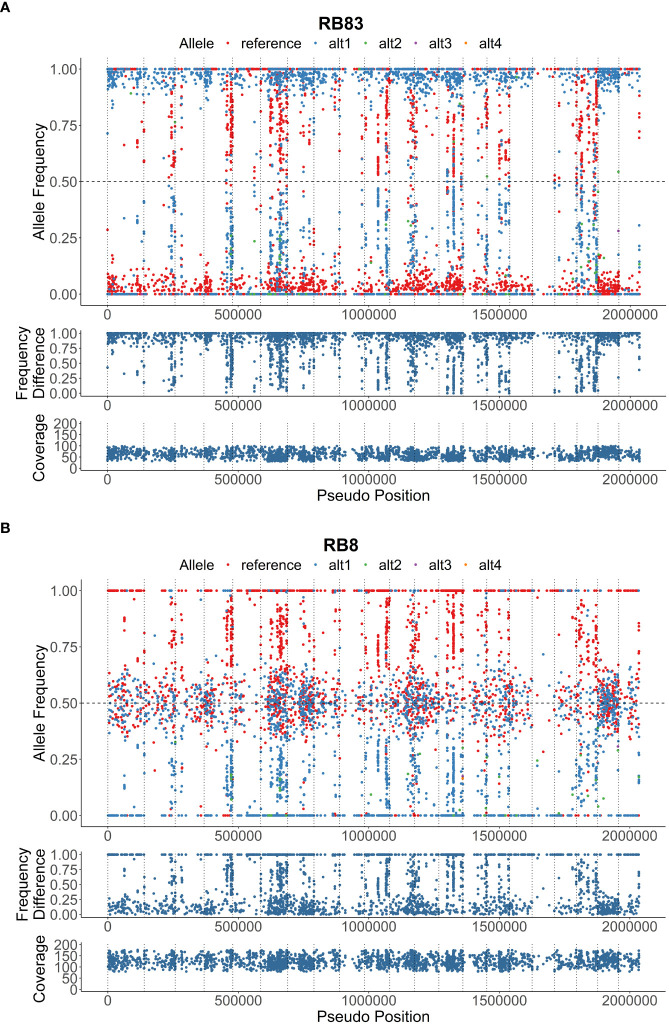
Heterozygous allele frequencies plots across 2 Mbp of the reference genome; **(A)**, isolate RB83 exhibiting low heterozygosity, and **(B)**, isolate RB8 exhibiting moderate heterozygosity. The allele frequency, frequency difference, and coverage are denoted on below the visualizations of heterozygous allele frequencies.

Genotypic diversity estimates of overall population of *P*. *rubi* was high (H = 3.22 and λ = 0.96) (**Table 2**). H and λ values of isolates grouped by cultivar was also high. Low genetic diversity (Hexp = 0.02) was observed among these isolates. The Hexp was lowest (0.011) in the isolates from ‘Meeker’ and was the highest (0.019) in the isolates from ‘Chemainus’ (**Table 2**). The SNPs per kb for isolates grouped by cultivar ranged from 0.60 to 1.70. Low heterozygous variants per kb were observed in 72% of pathogen isolates and the alternate allele coverage fell into a distribution of near 100% or 0% in these isolates (**Figure 1A**). Seven isolates, recovered from ‘Rudi’, showed signs of increased heterozygosity (SNPs per kb: 1.57 to 1.70; **Figure 1B**). The r̄_d_ values for the overall population and oomycetes isolates grouped by cultivar were significantly deviated from hypothesis of random mating among isolates of *P. rubi* from BC (**Table 2**).

**Table 2:** Indices of genotypic diversity for *Phytophthora rubi* from cultivar population based on SNPs generated by whole genome resequencing.

Population	N^a^	H^b^	λ^c^	Hexp^d^	r̄_d_^e^
‘Chemainus’	12	2.48	0.92	0.019	0.40***
‘Meeker’	3	1.09	0.67	0.011	0.03***
‘Rudi’	9	2.19	0.89	0.018	0.29***
Total	24	3.22	0.96	0.020	0.2061***

^a^N, number of individual isolate samples. ^b^H, Shannon-Wiener genotypic diversity. ^c^λ, Simpson’s diversity index. ^d^Hexp, Nei’s unbiased gene diversity. ^e^r̄_d_, Standardized index of association. *** P value 0.001.

**Figure 2:**
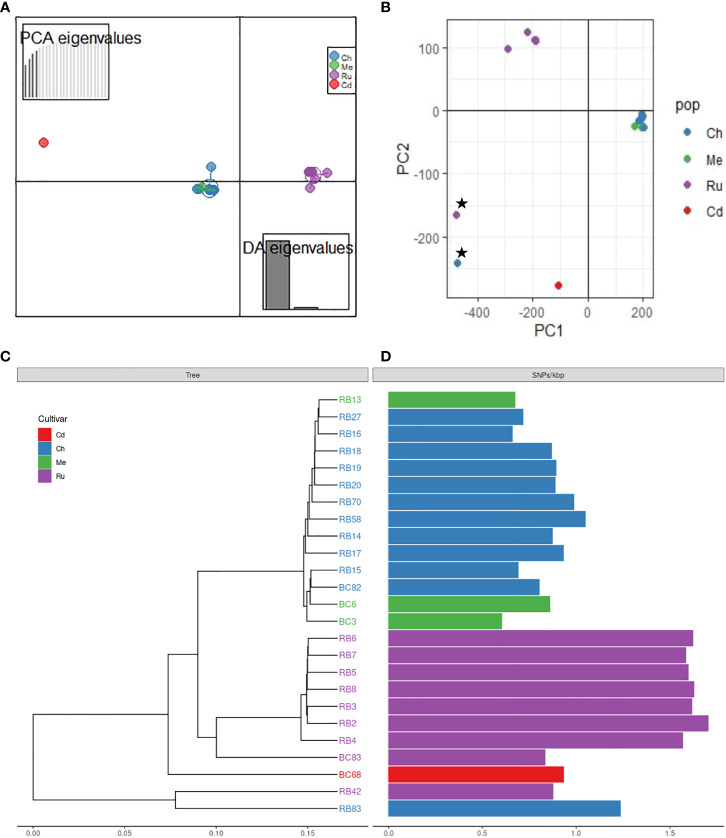
Population structure of *Phytophthora rubi* isolates from different cultivars of raspberry (cultivar abbreviation: Ru = ‘Rudi’, Ch = ‘Chemainus’, Me = ‘Meeker’, Cd = ‘Cascade Delight’) based on SNPs generated by whole genome resequencing; **(A)** discriminant analysis of principal components (DAPC) plot, **(B)** principal component analysis (PCA) plot; star symbols indicate outlier isolates RB83 and RB42, recovered from cultivar ‘Chemainus’ and ‘Rudi’, **(C)** genetic distance tree, **(D)** single nucleotide polymorphisms (SNPs) per Kbp.

DAPC indicated the presence of unique genetic clusters of *P rubi* isolates corresponding to raspberry cultivars (**Figure 2A**). Analysis of isolates using PCA based on Bruvo’s distance also showed the major grouping of isolates based on cultivars (**Figure 2B**). A phylogenetic tree also supported the results obtained from DAPC and PCA analyses (**Figure 2C**). Among the isolates of *P. rubi*, eight isolates from ‘Rudi’ formed one cluster and 11 isolates from ‘Chemainus’ and three isolates from ‘Meeker’ formed a distinct cluster.

## Discussion

This is the first genome-level study of *P. rubi* isolates collected from diverse cultivars and locations of raspberry growing areas in BC, Canada. Genome assemblies of 24 P*. rubi* isolates were generated along with gene predictions and annotation. BUSCO analysis found at least 92% of single-copy eukaryotic genes in the predicted proteins from each of the sequenced genomes, indicating that the gene prediction has captured a relatively complete representation of the gene content of each isolate. BUSCO values from our study are similar to results for the Eukarya dataset for previous *Phytophthora* genome assemblies studies produced from both short-read and long-read sequences (Cui et al., 2019; Engelbrecht et al., 2021; Kronmiller et al., 2023). Previous researchers sequenced only one (Tabima et al., 2017) or three isolates (Adams et al., 2020) of *P. rubi* resulting in assemblies of 74.65 and 77.84 Mbp in 9,434 and 13,882 scaffolds, respectively. The genome assemblies and scaffolds of *P. rubi* isolates generated in our study ranged from 59.45 to 71.16 Mbp and 7,203 to 11,143, respectively (**Table 1**), indicating that the genome structure in our study varied slightly than the previous studies, which is likely due to the inclusion of multiple isolates of *P. rubi* collected from diverse cultivars and locations in our study compared to either one or few isolates in previous studies.

We found a high genotypic diversity and low gene diversity among isolates of *P. rubi* from BC. We hypothesize that genotypic variability could be associated with three possible reasons (Milgroom, 2015; Zhan, 2016; Brugman et al., 2022). First, the continuous availability of host to pathogen populations is likely to promote rapid coevolution between the *P. rubi* pathogen and its raspberry host. Second, high disease pressure of PRRW in the field as a result of multiple infection events could increase diversity. Third, the mutation and sexual reproduction (through self-fertilization) can affect genotypic variability. Previous studies also reported the high genotypic diversity in clonal populations of *P. infestans* in Denmark (Maurice et al., 2019), and *P. palmivora* in Indonesia (Brugman et al., 2022). In contrast, genetic structure study of *P. rubi* population in the western USA using amplified fragment length polymorphism (Stewart et al., 2014) and using genotyping by sequencing (Tabima et al., 2018) showed low genetic diversity among isolates.

*Phytophthora rubi* is thought to be homothallic like other *Phytopthora* species (Tabima et al., 2018; Abad et al., 2023), with sexual reproduction through selfing and rare outcrossing. Selfing is expected to dramatically reduce heterozygosity and may reduce genetic diversity and thus considerably decrease the level of heterozygosity relative to sexual populations. Thus, we hypothesized that in *P. rubi* population very low heterozygous sites per Kb should be present in the genome. Our findings reveal the significant deficit of heterozygosity in the majority of sequenced genomes (18 of 25 isolates = 72%) indicating that the *P. rubi* population from BC are predominantly clonal (**Figure 2D**). However, the remaining seven isolates showed signs of increased heterozygosity, and these isolates were recovered from the raspberry cultivar ‘Rudi’. It is likely due to the accumulation of mutations during the successive asexual reproduction but may be due to a rare outcrossing event (Balloux et al., 2003). Nevertheless, our findings of clonality is congruent with the study in western USA by (Tabima et al., 2018) who also reported that *P. rubi* from raspberry are predominantly clonal and/or selfing. Previous studies also revealed low heterozygosity among populations of other homothallic *Phytopthhora* species, such as *P. plurivora* (Schoebel et al., 2014), and *P. sojae* (Stewart et al., 2016).

The production of oospores by pathogen does not always refer to sexual reproduction or its contribution to epidemics, hence it is important to test the null hypothesis of random mating when interpreting population structure of *P. rubi*. The r̄_d_ test showed the presence of clonality among isolates of *P. rubi* from BC. Past studies also used r̄_d_ to refer to clonality in populations of *P. pluvialis* (Brar et al., 2018), and *P. plurivora* (Schoebel et al., 2014). It is important to remember that the pathogen population dynamics would change over time and continuous monitoring of structure of pathogen populations is required to provide valuable information for breeders to identify resistant plant materials (McDonald, 2015; Milgroom, 2015). For example, Engelbrecht et al. (2017) reported clonal mode of reproduction in *P. cinnamomi*, a causal agent of Avocado root rot, populations in South Africa; however after a few years an evidence of random mating among pathogen populations was reported (Engelbrecht et al., 2022).

DAPC, PCA, and phylogenetic analyses revealed the presence of distinct clusters of *P. rubi* isolates with raspberry cultivars. The isolate from ‘Rudi’ formed one cluster while isolates from ‘Chemainus’ and ‘Meeker’ formed a further distinct cluster. This clustering may suggest that the genetic diversity in cultivars could pose varying levels of selection pressure on *P. rubi* populations (Moore and Daubeny, 1993; Moore, 2004; Kempler et al., 2006). We further speculate that the ability of variety of isolates of *P. rubi* to cause PRRW disease on a different raspberry cultivars might have led for cultivar specificity among isolates. Studies of 11 isolates of *P. fragariae* var *rubi*, (recently separated into a distinct species *P. rubi*) on roots of six raspberry genotypes showed that the existence of three different races, suggesting a significant cultivar/isolate interaction (Kennedy and Duncan, 1993). The presence of physiological races among pathogen populations was reported from other *Phytophthora* spp. such as *P. capsici* (Jiang et al., 2015; Barchenger et al., 2018) and *P. sojae* (Xue et al., 2015; Ping et al., 2016). Further research would be required to fully understand the pathogen races status of *P. rubi* isolates from BC using cultivars with varying levels of resistance.

Overall, this study provided the genome sequence resources of economically important raspberry pathogen *P. rubi* and investigated the genome structure and genetic diversity among these isolates. Knowledge gained from the genome sequences can provide insight into pathogen genomic structure, biology, comparative genomic and evolutionary studies among oomycetes. More recently, researcher have been using whole genome sequence data in examining effector biology and in comparative genomic analysis of different *Phytophthora* species including *P. rubi* and *P. fragariae* (Adams et al., 2020). The genome structure and diversity of pathogen populations could be associated with origin, dispersal of pathogen, and reproductive strategies (Milgroom and Peever, 2003; McDonald, 2015; Milgroom, 2015; Grünwald et al., 2017). This information could be useful for screening and developing resistant cultivars, and deploying disease management strategies. For example, pathogen microorganisms showing sexual or mixed reproduction could pose a high risk by emerging new genotypes during the sexual cycle (McDonald and Linde, 2002). Larger sampling and analysis of population within and outside production regions is warrant to understand overall population diversity of *P. rubi* populations both regionally and globally.

## Data availability statement

The datasets presented in this study can be found in online repositories. NCBI BioProject accession number is PRJNA856328. The variant data file (VCF) is available in the European Variation Archive (https://www.ebi.ac.uk/eva/?Home) and accessions associated with the submission are Project: PRJEB62133 and Analyses: ERZ18306854.

## Author contributions

SS, RB, and KL conceived and designed the study. RB supervised the first author to conduct the experiment and drafting the manuscript. SS, RB, and KL curated the data. SS and ML performed all bioinformatics analyses. SS and RB wrote a first draft of the manuscript. All authors read and approved the submitted version.
