# Supplementary material for: Genome resources and whole genome resequencing of Phytophthora rubi isolates from red raspberry
Source: Front Plant Sci. 2023 Jun 29;14:1161864. doi: 10.3389/fpls.2023.1161864 (PMC10339809; doi:10.3389/fpls.2023.1161864)
Supplement: Supplementary file 1 [file Table_1.docx]

**Supplementary Table 1 |** Summary of BUSCO statistics for genome resequencing of 24 isolates of *Phytophthora rubi*.

| **Isolate** | **Cultivar** | **Year** | **Field** | **Location** | **Total sequencing yield** | **Assembler** | **BUSCO completeness** | **Single Copy BUSCO genes** | **Duplicated BUSCO genes** | **Fragmented BUSCO genes** | **Missing BUSCO genes** |
| --- | --- | --- | --- | --- | --- | --- | --- | --- | --- | --- | --- |
| BC83 | ‘Rudi’ | 2019 | F6 | Abbotsford | 10,44,54,967 | Spades | 92.60% | 87.50% | 5.10% | 2.00% | 5.40% |
| RB2 | ‘Rudi’ | 2020 | F6 | Abbotsford | 9,88,85,509 | Abyss | 93.70% | 87.80% | 5.90% | 2.00% | 4.30% |
| RB3 | ‘Rudi’ | 2020 | F6 | Abbotsford | 7,81,49,065 | Abyss | 92.60% | 87.10% | 5.50% | 2.40% | 5.00% |
| RB4 | ‘Rudi’ | 2020 | F6 | Abbotsford | 8,88,37,474 | Abyss | 93.80% | 87.50% | 6.30% | 2.00% | 4.20% |
| RB42 | ‘Rudi’ | 2020 | F19 | Abbotsford | 14,20,37,105 | Abyss | 92.20% | 85.90% | 6.30% | 2.40% | 5.40% |
| RB5 | ‘Rudi’ | 2020 | F6 | Abbotsford | 7,41,11,127 | Abyss | 94.10% | 88.20% | 5.90% | 1.60% | 4.30% |
| RB6 | ‘Rudi’ | 2020 | F6 | Abbotsford | 9,01,36,727 | Abyss | 92.60% | 86.70% | 5.90% | 2.40% | 5.00% |
| RB7 | ‘Rudi’ | 2020 | F6 | Abbotsford | 8,82,12,303 | Abyss | 93.40% | 87.10% | 6.30% | 2.70% | 3.90% |
| RB8 | ‘Rudi’ | 2020 | F6 | Abbotsford | 8,07,50,986 | Abyss | 93.00% | 87.10% | 5.90% | 2.00% | 5.00% |
| BC82 | ‘Chemainus’ | 2019 | F23 | Chilliwack | 1,13,191,754 | Abyss | 94.20% | 87.50% | 6.70% | 1.60% | 4.20% |
| RB14 | ‘Chemainus’ | 2020 | F9 | Abbotsford | 9,34,55,783 | Abyss | 93.00% | 87.10% | 5.90% | 2.40% | 4.60% |
| RB16 | ‘Chemainus’ | 2020 | F9 | Abbotsford | 10,07,51,340 | Abyss | 93.80% | 87.50% | 6.30% | 2.00% | 4.20% |
| RB17 | ‘Chemainus’ | 2020 | F9 | Abbotsford | 4,10,37,298 | Abyss | 93.30% | 88.20% | 5.10% | 2.70% | 4.00% |
| RB18 | ‘Chemainus’ | 2020 | F9 | Abbotsford | 4,30,34,453 | Spades | 93.70% | 88.60% | 5.10% | 1.60% | 4.70% |
| RB19 | ‘Chemainus’ | 2020 | F9 | Abbotsford | 4,59,71,739 | Spades | 93.30% | 87.80% | 5.50% | 2.00% | 4.70% |
| RB20 | ‘Chemainus’ | 2020 | F9 | Abbotsford | 4,05,58,261 | Spades | 93.00% | 87.50% | 5.50% | 2.00% | 5.00% |
| RB27 | ‘Chemainus’ | 2020 | F17 | Abbotsford | 17,58,60,947 | Abyss | 94.10% | 88.20% | 5.90% | 1.60% | 4.30% |
| RB58 | ‘Chemainus’ | 2020 | F23 | Chilliwack | 4,55,93,604 | Spades | 93.00% | 87.10% | 5.90% | 2.70% | 4.30% |
| RB70 | ‘Chemainus’ | 2020 | F25 | Agassiz | 3,85,80,918 | Spades | 93.70% | 88.20% | 5.50% | 2.00% | 4.30% |
| RB83 | ‘Chemainus’ | 2020 | F28 | Abbotsford | 3,53,71,154 | Spades | 93.40% | 87.50% | 5.90% | 2.00% | 4.60% |
| BC3 | ‘Meeker’ | 2018 | F7 | Abbotsford | 9,34,76,419 | Spades | 93.40% | 87.50% | 5.90% | 2.00% | 4.60% |
| BC6 | ‘Meeker’ | 2018 | F7 | Abbotsford | 11,21,58,902 | Abyss | 93.00% | 86.70% | 6.30% | 2.40% | 4.60% |
| RB13 | ‘Meeker’ | 2020 | F7 | Abbotsford | 10,12,00,368 | Abyss | 93.40% | 87.50% | 5.90% | 2.00% | 4.60% |
| BC68 | ‘Cascade Delight’ | 2019 | F22 | Delta | 10,89,70,365 | Spades | 93.70% | 88.20% | 5.50% | 2.00% | 4.30% |
